# Establishment of perpendicular protrusion of type I collagen on TiO_2_ nanotube surface as a priming site of peri-implant connective fibers

**DOI:** 10.1186/s12951-019-0467-1

**Published:** 2019-03-01

**Authors:** Toshiki Nojiri, Chia-Yu Chen, David M. Kim, John Da Silva, Cliff Lee, Masahiko Maeno, Arthur A. McClelland, Bryan Tse, Shigemi Ishikawa-Nagai, Wataru Hatakeyama, Hisatomo Kondo, Masazumi Nagai

**Affiliations:** 1 Department of Oral Medicine, Infection and Immunity, Harvard School of Dental Medicine, 188 Longwood Avenue, Boston, MA USA; 2 Department of Restorative Dentistry and Biomaterials Sciences, Harvard School of Dental Medicine, 188 Longwood Avenue, Boston, MA USA; 3 Department of Adhesive Dentistry, The Nippon Dental University, 1-9-20 Fujimi, Chiyoda-ku, Tokyo, Japan; 4 Center for Nanoscale Systems, Harvard University, 11 Oxford St, Cambridge, MA USA; 5 Department of Prosthodontics and Oral Implantology, School of Dental Medicine, Iwate Medical University, 19-1 Uchimaru, Morioka, Iwate Japan

**Keywords:** Implant surface, Titanium, Nanotubes, Collagen, Perpendicular orientation

## Abstract

Natural teeth are supported by connective tissue collagen fibers that insert perpendicularly in the tooth cementum. Perpendicular insertion plays an important role in the maintenance of the junction between the oral epithelium and the periodontal connective tissue. Most titanium dental implant surfaces have no micro or macro structure to support perpendicularly oriented collagen attachment. Without this tight biologic seal to resist bacterial invasion and epithelial downgrowth, progressive bone loss in peri-implantitis is seen around dental implants. The purpose of this study was to establish the perpendicularly oriented collagen attachment to titanium oxide nanotube (TNT), and to assess its binding stability. TNT was prepared on the titanium-surface by anodization. Scanning electron microscopy (SEM) showed a regularly aligned TNT with an average 67 nm-diameter when anodized at 30 V for 3 h. Subsequently, collagen type I (CoI) was electrophoretically fused to anodic TNT in native polyacrylamide gel system where negatively charged CoI-C term was perpendicularly navigated to TNT. SEM and atomic force microscopy (AFM) were used to analyze CoI on the TiO_2_ and TNT surface. Several tens of nanometers of CoI protrusion were recorded by AFM. These protrusions may be long enough to be priming sites for cell-secreted CoI. CoI laid parallel to the titanium surface when fused by a chemical linker. Binding resistance of CoI against drastic ultrasonication was measured by Fourier-transform infrared spectroscopy attenuated total reflection (FTIR-ATR). The electrophoretically fused CoI in the titanium nanotube (TNT–CoI^EPF^) showed the significantly greatest binding resistance than the other groups (P < 0.01, a 1-way ANOVA and Tukey HSD post hoc test). Furthermore, TNT–CoI^EPF^ surface rejected epithelial cell stretching and epithelial sheet formation. Chemically linked horizontal CoI on titanium oxide (TiO_2_) facilitated epithelial cell stretching and sheet formation.

## Introduction

Although a dental implant may be the treatment choice for fixed prosthodontics for partially edentulous or edentulous patients, implant-related complications are frequently reported [1]. One of the major biological complications affecting osseointegrated implants is peri-implantitis, a bacterial plaque-associated pathological condition characterized by inflammation of peri-implant tissue sand subsequent progressive loss of supporting bone [2–4]. Similar to periodontitis, peri-implantitis is caused by the inflammatory response to bacterial plaque, such as *Porphyromonas gingivalis* and *Tannerella forsythia* [5, 6]. There is a significant difference between the soft tissue interface of natural teeth and dental implants. The junctional epithelium (JE) in healthy dentition attaches to the tooth surface with basal lamina (BL) at the cervical level, and perpendicular attachment of dentogingival fibers block the downgrowth of the epithelium [7]. The perpendicular insertion of the gingival and periodontal ligament (PDL) fibers into the cementum plays the critical role in maintaining the vertical level of the epithelial attachment and flexible suspension of the teeth. In contrast, bundles of collagen fibers run parallel, circumferentially around the implant surface, and are not able to resist the epithelial downgrowth [8, 9]. Moreover, peri-implant JE faces the implant at the apical side of the cell with no BL adherent apparatus [10–12]. The combination of weak epithelial attachment creating a vulnerable pocket for biofilm invasion and lack of perpendicular connective tissue attachment to resist further downgrowth allows for rapid and progressive destruction in peri-implantitis. A next generation of dental implant with BL-mediated JE attachment supported by perpendicular connective tissue attachment at the implant/abutment interface is needed.

Perpendicular attachment onto titanium has been reported for laser-machined horizontal microgrooves that limits the direction of cell division to the horizontal plane [13–17]. Other research employed activated platelets on the titanium surface to induce functional epithelial attachment via basal lamina to resist bacterial invasion [18, 19]. A long-lasting vertical level of the epithelial attachment with an ideal biologic seal might be achieved when the above-mentioned technologies are combined with the nanotubes and collagen fibers to modify the implant surface.

Titanium oxide nanotubes (TNT), in particular, have become increasingly popular due to the simplicity and low cost to fabricate this surface [20–23]. Multiple studies have employed TNT as a scaffold for the delivery of bioactive molecules that can enhance dental implant osseointegration [24–26]. However, no thorough investigation has been conducted on the potential use of the nanoscale structure for the attachment of peri-implant collagen fibers. We focused our attention on the size and structure of the TNT as a potential seeding vessel for triple helix monomers of collagen type I (CoI). Diameter and depth of TNT can be sized around several 10 to 100 nanometers to which CoI monomers of 1.5 nm in diameter and 300 nm in length can be planted perpendicularly. Owing to the phenomenon that negatively-charged triplet C-terminus of CoI align at the same side, we postulated that CoI monomers could be perpendicularly induced into TNT as an anode in an electrophoretic system.

The aims of this study were: (1) to establish the electrophoretic method for the perpendicular implantation of CoI into TNT (CoI/TNT); (2) to assess the binding stability of CoI/TNT; and (3) to test epithelial adhesion on CoI/TNT to predict the inhibition of epithelial downgrowth in vivo.

## Materials and methods

### Optimization of TNT formation on the titanium surface

**Fig. 1:**
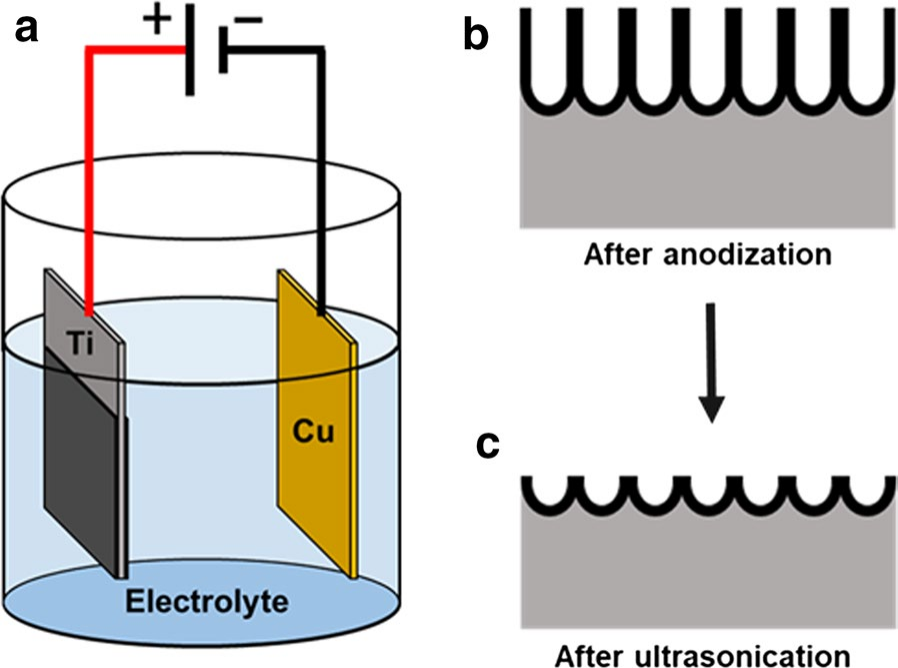
This scheme illustrates the electrochemical anodization process used to fabricate the TNT on titanium specimens. **a** The titanium pieces were used as anodes and the cupper pieces as cathode in a fluoride-based electrolyte solution. **b** After anodization, TNT was obtained. **c** TNT was removed with ultrasonication to unveil a glossy surface underneath with a uniform array of shallow TNT

We targeted the nanotubes with a 50 to 100 nm diameter which would be appropriate for the perpendicular insertion of 1.5 nm-diameter collagen monomers, and large enough for future collagen fibril insertion [27]. Anodization was applied to the titanium specimens (1.0 × 3.0 cm, 0.1 mm thickness, Grade-2 titanium, Gallium Source, LLC, CA, USA, Fig. 1). Copper was used as the cathode. Before anodization, titanium and copper samples were ultrasonically cleaned in 0.5% sodium dodecyl sulfate (SDS; Sigma, MO, USA), deionized water, acetone (Sigma, MO, USA) and ethanol (Sigma, MO, USA), sequentially, for 20 min in each solvent, and then air-dried. The distance between the anodic titanium and cathodic copper was 2.5 cm. The anodization was performed in an electrolyte solution of ammonium fluoride (NH_4_F) at 0.38 wt% and H_2_O at 1.79 wt% in ethylene glycol with constant voltages of 1, 5, 10, 20, 30 and 50 V for 1, 3 and 5 h at each voltage. Specimens were washed in two-step sonications with 30% H_2_O_2_ for 5 min first, and then in 0.1 M acetic acid for 60 min. After cleaning, the center of each titanium sample was cut into two 1.0 cm × 1.0 cm samples for scanning electron microscopy (SEM) (Zeiss Supra 55VP field emission scanning electron microscope; ZEISS, Oberkochen, Germany). Three regions of interest on the diagonal for each specimen were randomly selected. A number of clearly outlined TNT were counted using Image J (National Institutes of Health, MD, USA). Diameter of TNT were also calculated. The average of triplet of each of 18 conditions were used for the data analysis.

### Linking of CoI to titanium samples

**Fig. 2:**
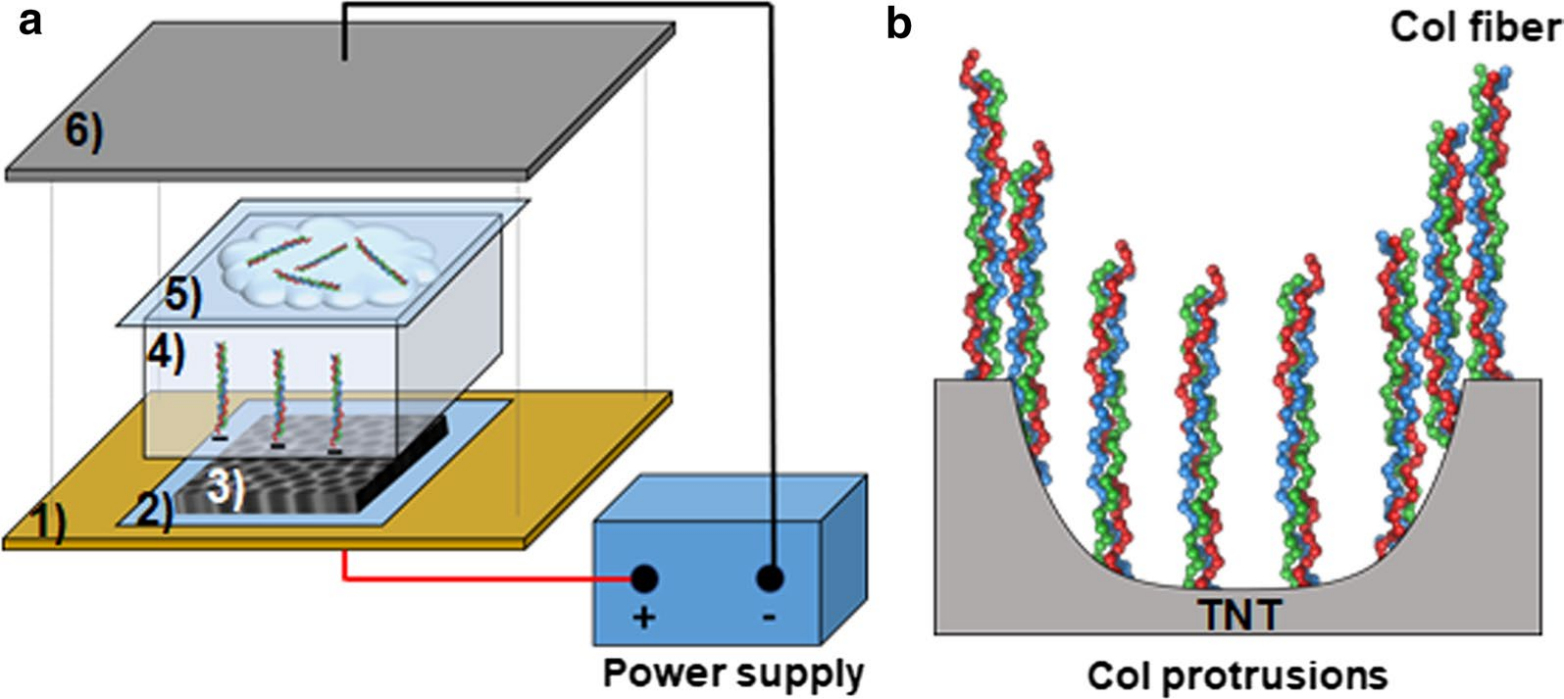
This scheme illustrates EPF and perpendicular CoI inserted into the TNT. **a** EPF: CoI was transferred onto TNT in a semi dry transfer. The transfer unit was assembled from the bottom in the following order: (1) anode of a semi dry blotter; (2) 1× TGB-wetted filter paper; (3) TNT surface upward; (4) 6% Bis Tris gel; (5) TGB-wetted filter to which CoI was spotted at the titanium facing area; (6) cathode. **b** Scheme of perpendicular CoI inserted into the TNT

CoI (Atelocollagen: Atelo Cell^®^ IPC-30, KOKEN, Japan) with a monomer length of 300 nm was fabricated to titanium oxide (TiO_2_) and TNT by means of electrophoretic fusion (EPF) and phosphonic acid (PA)-based chemical linking (CL). EPF was carried out in the semi-dry transfer system (Fig. 2). The transfer unit was assembled in the following order from the bottom: (1) anode of a semi dry blotter (Trans-Blot Turbo Transfer System^®^, BIO-RAD Laboratory, CA, USA); (2) 1× tris glycine buffer (TGB)-wetted filter paper; (3) TiO_2_ or TNT surface upward of titanium; (4) 6% Bis Tris gel (BIO RAD Laboratory); (5) CoI-spotted TGB-wetted filter; and (6) cathode. CoI was diluted at 0.3 mg/mL in TGB before spotted on the filter. CoI was run at constant voltage of 25 V for 2 min. After the transfer, the specimens were washed 3 times with PBS.

Phosphonic acid-based chemical linker with amide-end (10-CDPA; Dojindo Molecular Technology) was used for CL-method as reported by Sugawara et al. [18]. Briefly 10-CDPA chemically bound to TiO_2_ at phosphonic acid-residues, and CoI was coupled to the amide-end by the EDC/NHS-chemistry.

### Imaging analyses of CoI-fused titanium surfaces

The molecular topology of CoI on titanium was analyzed with an atomic force electron microscopy (AFM) (Cypher AFM, Asylum Research, CA, USA). A part of the CoI applied titanium was fixed in 4% paraformaldehyde (PFA), washed in water, and dehydrated in ethanol series to 100% (75%, 80%, 85%, 90%, 95% and 100%, 20 min in each incubation) for SEM.

### Binding stabilities of CoI to titanium surfaces

Binding stabilities of CoI to different titanium surfaces were evaluated with a mechanical wash test. Tested specimens included the combination of two binding methods, EPF and CDPA-CL and two titanium surfaces of TNT and TiO_2_: TiO_2_ linked collagen through CL (TiO_2_–CoI^CL^), TNT linked collagen through CL (TNT–CoI^CL^), TiO_2_ fused collagen through EPF (TiO_2_–CoI^EPF^) and TNT fused collagen through EPF (TNT–CoI^EPF^). The specimens were sonicated in PBS with an ultrasonic disruptor (VWR Branson 250 Sonifier, MA, USA). The distance between the specimen and the ultrasonic probe tip was fixed at 10 cm. One cycle was 15 s at maximum power. The procedure was repeated for 3 and 10 cycles in each group with a 45 s pause between cycles.

CoI-binding at titanium surface was analyzed by Fourier Transform Infrared Spectroscopy Attenuated Total Reflection (FTIR-ATR) (Lumos FTIR Microscope, Bruker, MA, USA). The ATR crystal was a single bounce germanium crystal with 125-micron diameter. The treated titanium samples were examined using FTIR-ATR with 4 cm^−1^ resolution. Sixteen scans were averaged together for each data point over the range of 600–4000 cm^−1^.

Following the FTIR-ATR peak assignments in the literature [28–30], the existence of CoI and its binding strength to the titanium surface were determined by the backbone collagen amide I vibrations (C=O stretching vibration absorption peak: near 1651 cm^−1^) and amide II vibrations (N–H stretching vibration absorption peak: near 1519 cm^−1^) and hydrogen bond vibrations (Ti–OH absorption peak: 1089 cm^−1^).

The data was processed by subtracting the FTIR-ATR spectra of the control titanium sample, from the respective test samples. The resulting difference spectra were baseline-corrected. The peak height of the absorbance peak corresponding to the amide I, amide II, and Ti–OH bonds of the FTIR-ATR spectrum were calculated using OPUS software.

### Cell attachment

Attachment of epithelial cells to the modified TiO_2_ surface was evaluated as attached number and mode using a cell counting kit (CCK8, Dojindo molecular technology, WA, USA) and SEM, respectively. Human gingival epithelial cell line OBA9 cells (Professor Murakami at Osaka University, Japan) were maintained in keratinocyte-SFM medium (Thermo Fisher Scientific) at 37 °C in 5% CO_2_ and 95% atmospheric air. Cells were seeded on TiO_2_, TNT, TiO_2_–CoI^CL^, TNT–CoI^CL^, TNT–CoI^EPF^ at 100 k cells/cm^2^ in 200 µL of culture medium in each well of 48 well plate and settled in the culture for 3 h. Unbound cells were then gently washed off with the culture medium and the remaining cells were further cultured in 200 µL of culture medium supplemented with CCK8 reagent for another 3 h. One hundred microliter of CCK8 reaction was transferred to a 96-well plate for OD reading. Cells on each sample surface were washed with PBS and fixed in 4% PFA for SEM analysis.

### Statistical analysis

To compare the significant differences in the amount CoI on the tested titanium surfaces, one-way analysis of variance (ANOVA) was conducted for intragroup and intergroup relations. The average values of peak height in FTIR-ATR peaks of amide I, amide II, and Ti–OH bonds between groups were compared. The Tukey HSD post hoc test was applied to assess differences that were statistically significant. The significance level adopted was 5% for all tests.

## Results

### Optimization of TNT fabrication on the titanium surface

**Fig. 3:**
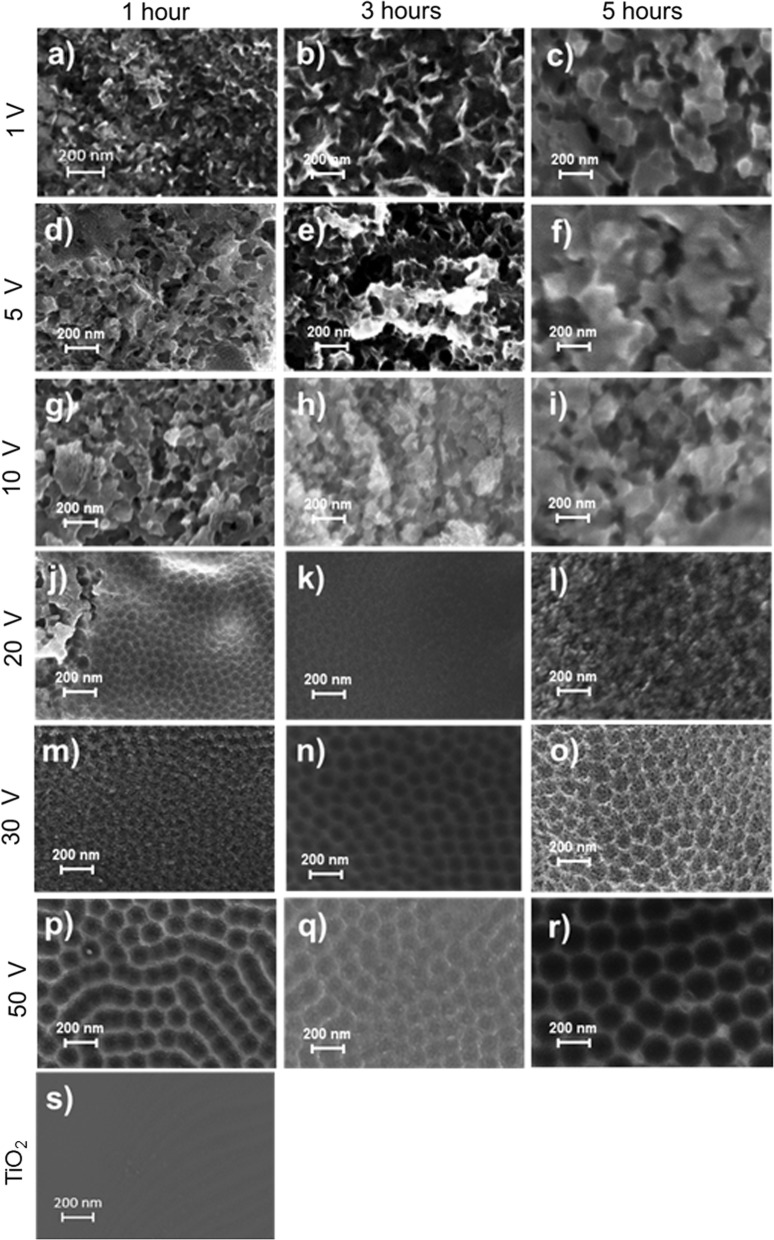
SEM images of titanium surface through anodization. **a** 1 V × 1 h, **b** 1 V × 3 h, **c** 1 V × 5 h, **d** 5 V × 1 h, **e** 5 V × 3 h, **f** 5 V × 5 h, **g** 10 V × 1 h, **h** 10 V × 3 h, **i** 10 V × 5 h, **j** 20 V × 1 h, **k** 20 V × 3 h, **l** 20 V × 5 h, **m** 30 V × 1 h, **n** 30 V × 3 h, **o** 30 V × 5 h, **p** 50 V × 1 h, **q** 50 V × 3 h, **r** 50 V × 5 h, **s** TiO_2_/control. Original magnification ×100,000

Regularly aligned array of TNT with 67 nm-diameter in average was observed in SEM when anodized at 30 V for 3 h (Fig. 3n). Under conditions with a lower voltage, nanotubes were not formed and yielded a rippled rough surface (Fig. 3a–i). At 20 V, regardless of duration, nanotubes were irregularly formed (Fig. 3j–l). Extended anodization at 50 V for 5 h yielded varied diameter TNT ranging from 100 to 150 nm (Fig. 3p–r). Voltage and duration dependent TNT-diameter and number per field under SEM at 10,000× are shown in Table 1. Based on the average diameter and the uniformity of TNT we concluded that 30 V and 3 h was the optimum combination of voltage and time.

**Table 1:** Measurement of nanotube number and diameter

Voltage (V)		0	1	5	10	20	30	50
Anodization (h)								
# of NTs observed	1 h	NF	NF	NF	NF	IF	IF	457.8 ± 30.5
	3 h	NF	NF	NF	NF	IF	601.3 ± 13.2	331.6 ± 32.9
	5 h	NF	NF	NF	NF	IF	350.9 ± 26.0	311.1 ± 29.8
Diameter (nm)	1 h	NF	NF	NF	NF	47.0 ± 3.7	66.4 ± 8.5	113.6 ± 8.6
	3 h	NF	NF	NF	NF	38.4 ± 3.0	67.0 ± 2.6	115 ± 5.3
	5 h	NF	NF	NF	NF	43.6 ± 3.0	68.3 ± 3.6	147.6 ± 11.7

# of TNT observed: View of magnification 100,000×

*NF* not formed, *IF* irregular formation

### CoI attachment

**Fig. 4:**
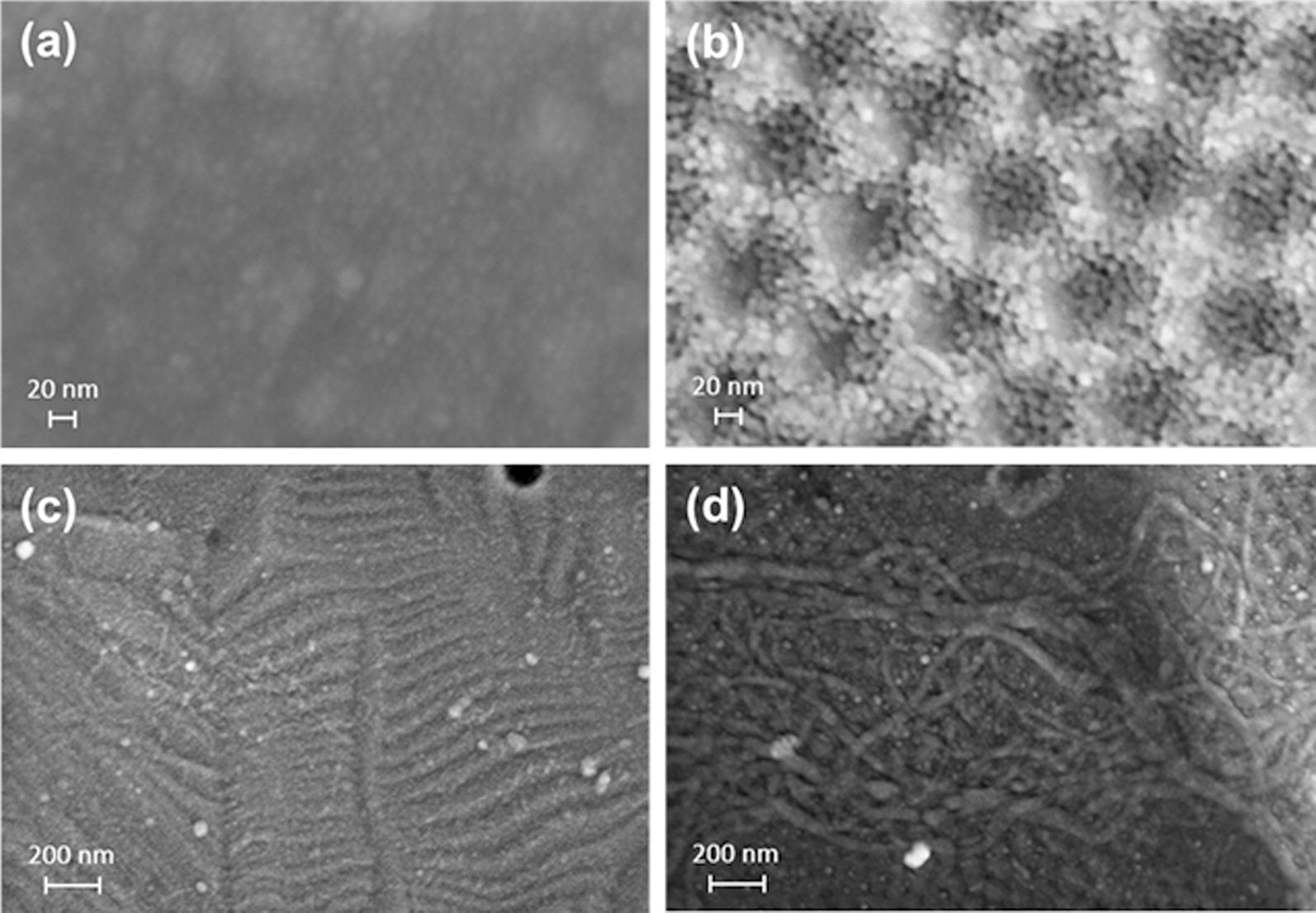
SEM images of CoI attached on the titanium surface. **a** TiO_2_–CoI^EPF^, **b** TNT–CoI^EPF^, **c** TiO_2_–CoI^CL^, and **d** TNT–CoI^CL^, **a**, **b** original magnification ×500,000. **c**, **d** original magnification ×100,000

Representative SEM images of CoI attachment via EPF and CL onto TiO_2_ and TNT surfaces are seen in Fig. 4. The overhead view of TiO_2_ surface in EPF groups showed CoI as nanodots indicating the perpendicular attachment on titanium surfaces. Nanodots of TNT–CoI^EPF^ were aligned on the TNT-edges and in the nanoholes (Fig. 4b) while poorly focused nanodots were seen on TiO_2_ (Fig. 4a). Diameter of each nanodot was between 5 and 10 nm suggesting perpendicular multimers of CoI. In CL groups, CoI was apparently laid down parallel to the long axis on both TiO_2_ (Fig. 4c) or TNT (Fig. 4d) surfaces.

**Fig. 5:**
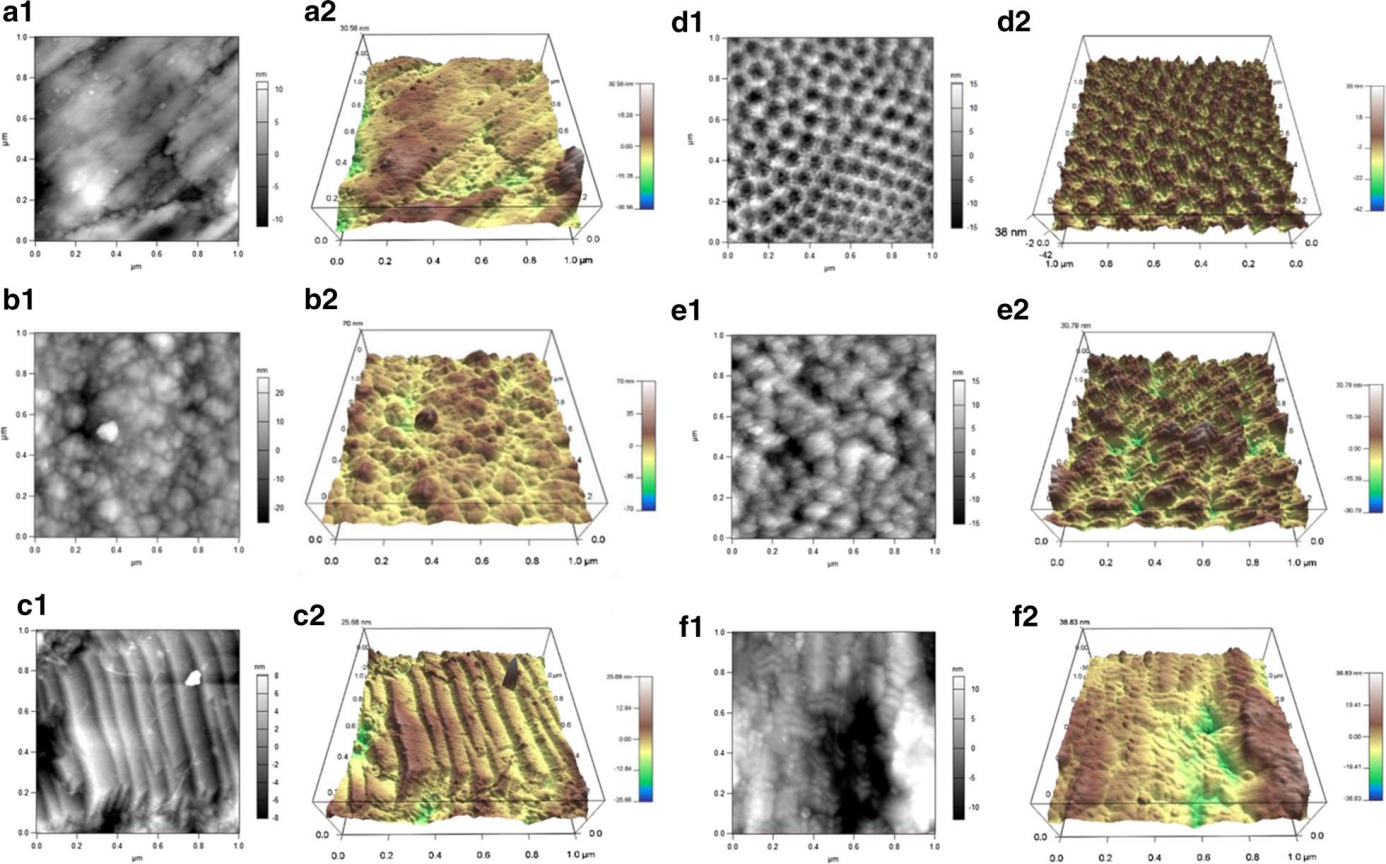
Surface topographic image through AFM for **a1** TiO_2_, **a2** 3D image of TiO_2_, **b1** TiO_2_–CoI^EPF^, **b2** 3D image of TiO_2_–CoI^EPF^, **c1** TiO_2_–CoI^CL^, **c2** 3D image of TiO_2_ –CoI^CL^, **d1** TNT, **d2** 3D image of TNT, **e1** TNT–CoI^EPF^, **e2** 3D image of TNT–CoI^EPF^, **f1** TNT–CoI^CL^, **f2** 3D image of TNT–CoI^CL^

Surface topography of different TiO_2_ and CoI attachment taken by AFM are shown in Fig. 5. While slight roughness was probed on TiO_2_ (Fig. 5a1, a2), a regularly aligned array of nanotubes was imaged in the anodization of 30 V for 3 h (Fig. 5d1, d2). On the TiO_2_-EPF surface, an amorphous nano-sized dot structure which is similar to the SEM image from Fig. 4a was observed (Fig. 5b2). On the TNT–CoI^EPF^ surface, the collagen fibers protrude perpendicularly from the nanotube surface, following the contour of the nanotubes underneath (Fig. 5e1, e2), and is remarkedly different than the AFM image of TNT (Fig. 5d1, d2), indicating that collagen was inserted perpendicularly to the nanotube surface. In the CL groups, similar to SEM images (Fig. 4c, d), collagen was laid down parallel to the long axis on both TiO_2_ and TNT surfaces (Fig. 5c1, c2, f1, f2).

### Binding stability of CoI

**Fig. 6:**
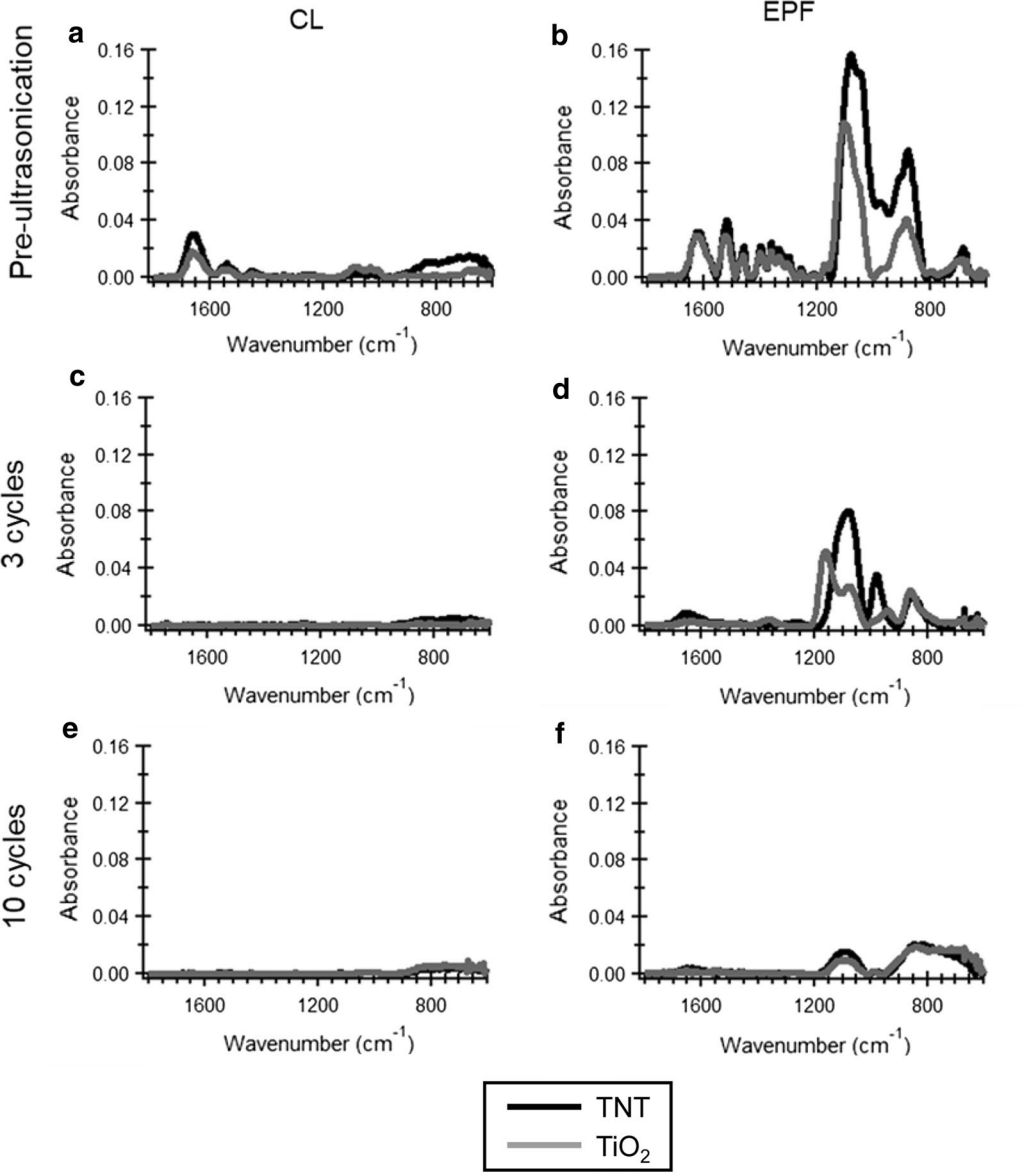
FITR-ATR spectra of CoI attached titanium surfaces with and without the ultrasonication. **a** Pre ultrasonication of CL group. **b** Pre ultrasonication of EPF group. **c** 3 cycles ultrasonication of CL group. **d** 3 cycles ultrasonication of EPF group. **e** 10 cycles ultrasonication of CL group. **f** 10 cycles ultrasonication of EPF group

**Fig. 7:**
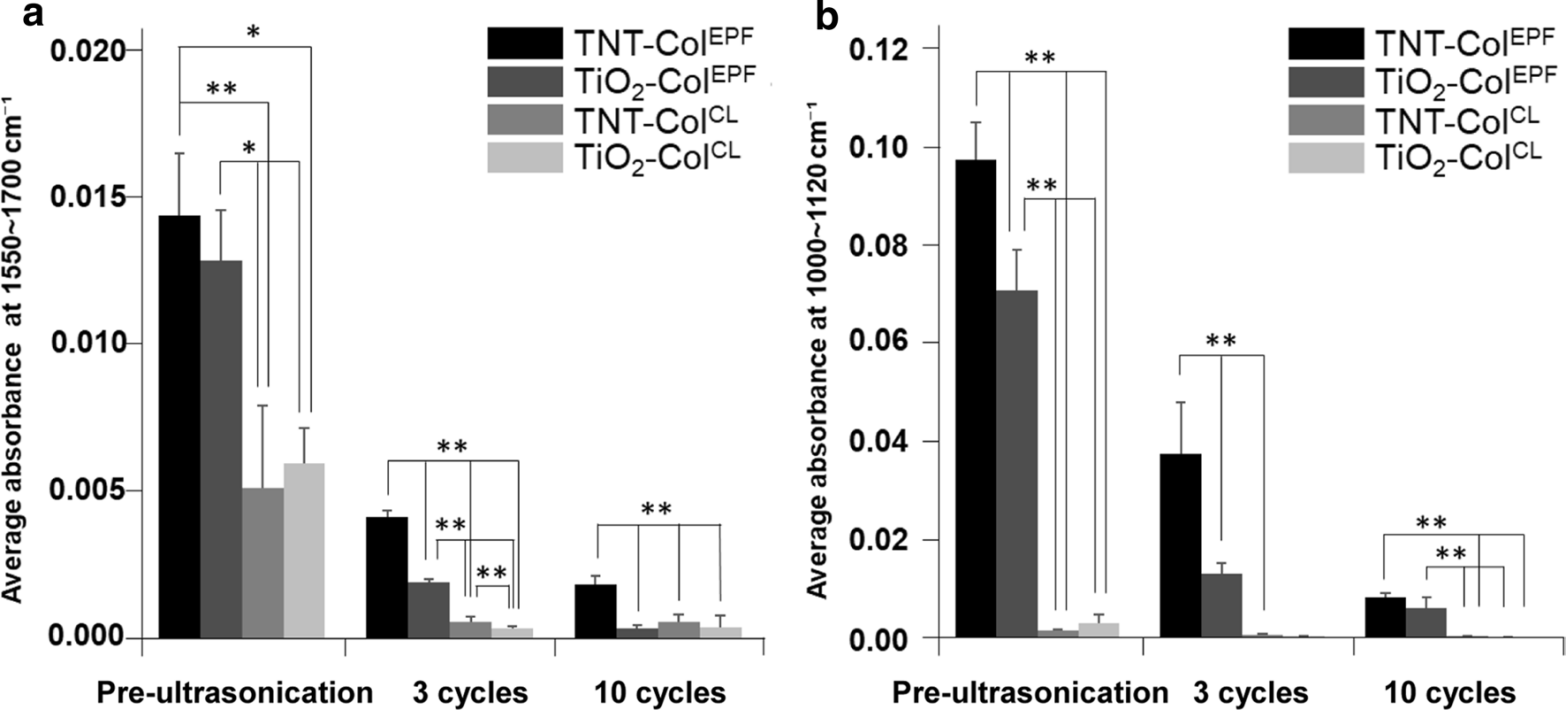
Average absorbance of the Amide bond and Ti–OH bond in FTIR-ATR spectra. **a** Average absorbance at 1550–1700 cm^−1^ corresponding amido bond. **b** Average absorbance at 1000–1120 cm^−1^ corresponding Ti–OH bond. Error bars represent mean ± SD for n = 3, *P < 0.05, **P < 0.01

CoI associated spectra were analyzed by FTIR-ATR to compare the binding stability on the titanium surfaces (Fig. 6). The Ti–OH absorption peak at 1089 cm^−1^ and the amide I bond at 1651 cm^−1^ were clearly identified after EPF on both TiO_2_ or TNT surfaces. After 3 sonication cycles, amide and Ti–OH bond peaks remained at 50% in TNT–CoI^EPF^ and 10% in TiO_2_–CoI^EPF^. The difference between the two groups was statistically significant (Fig. 7, P < 0.01, a 1-factor ANOVA and Tukey HSD post hoc test). These peaks disappeared in CL groups (Fig. 6c) in the first 3 cycles of sonication. In TNT–CoI^EPF^, the bond peaks still remained significant at higher than 10%. Similar trends in the reductions in the amide I and Ti–OH bonds were seen.

### Cell attachment to titanium surface

**Fig. 8:**
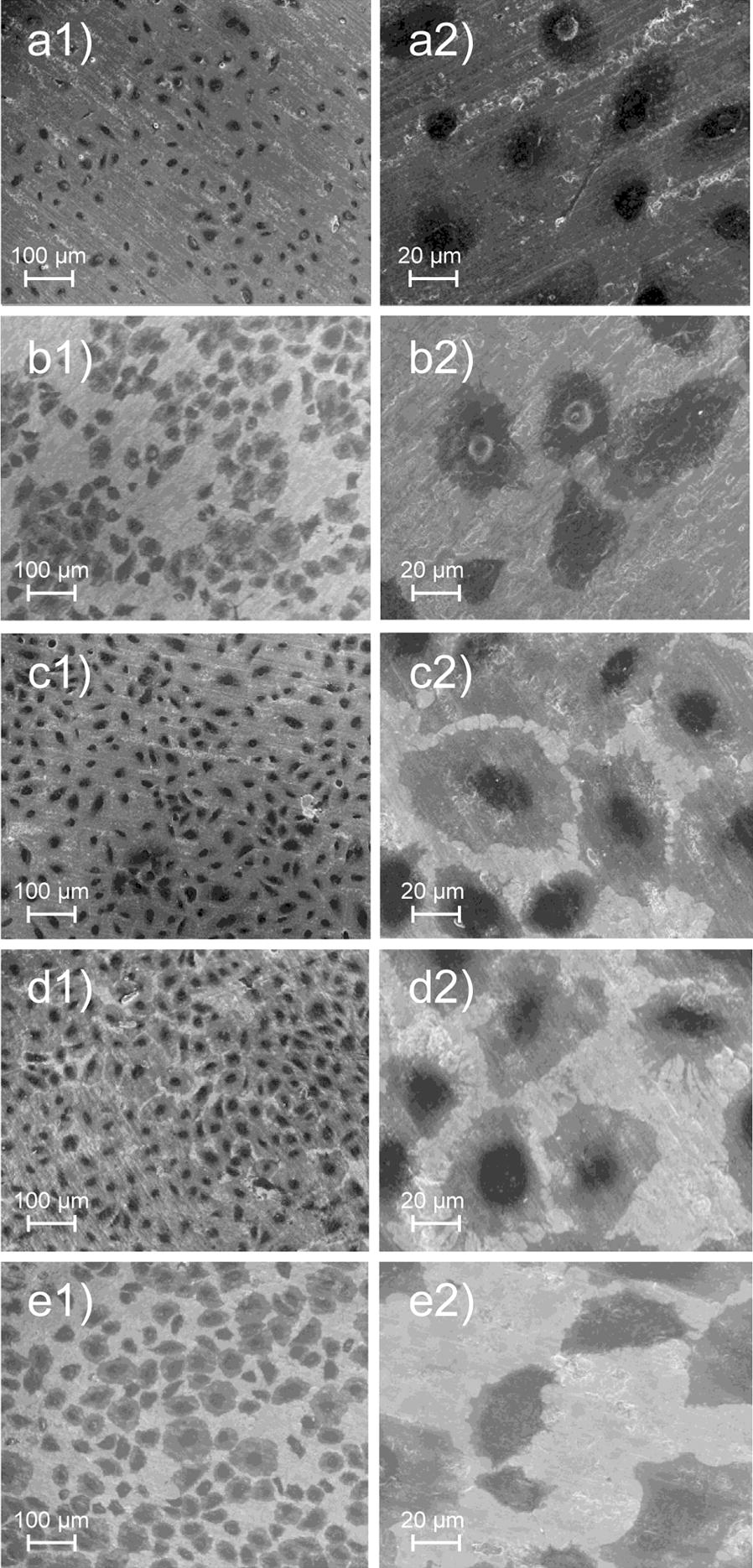
SEM images of epithelial cells attached on the surface of each group in cell attachment assay. **a1**,** 2** TiO_2_, **b1**, **2** TNT, **c1**, **2** TiO_2_–CoI^CL^, **d1**, **2** TNT–CoI^CL^, **e1**,** 2** TNT–CoI^EPF^. **a1**–**e1**: original magnification ×300. **a2**–**e2**: Original magnification ×1500

The mode of epithelial attachment on each treated titanium surface was identified by SEM ultrastructure (Fig. 8). On TiO_2_ and TNT surfaces without CoI, limited numbers of epithelial cells were seen (Fig. 8a1, b1). In addition, these cells exhibited round-up morphology of weak adhesion (Fig. 8a2, b2). On the other hand, on TiO_2_ and TNT surfaces with CL-mediated parallel CoI coating, cells with widely spread plasma membrane occupied the surface (Fig. 8c1, 2, d1, 2). On the TNT with perpendicular nanodot CoI surface, round up epithelial cells were sparsely seen (Fig. 8e1, 2).

**Fig. 9:**
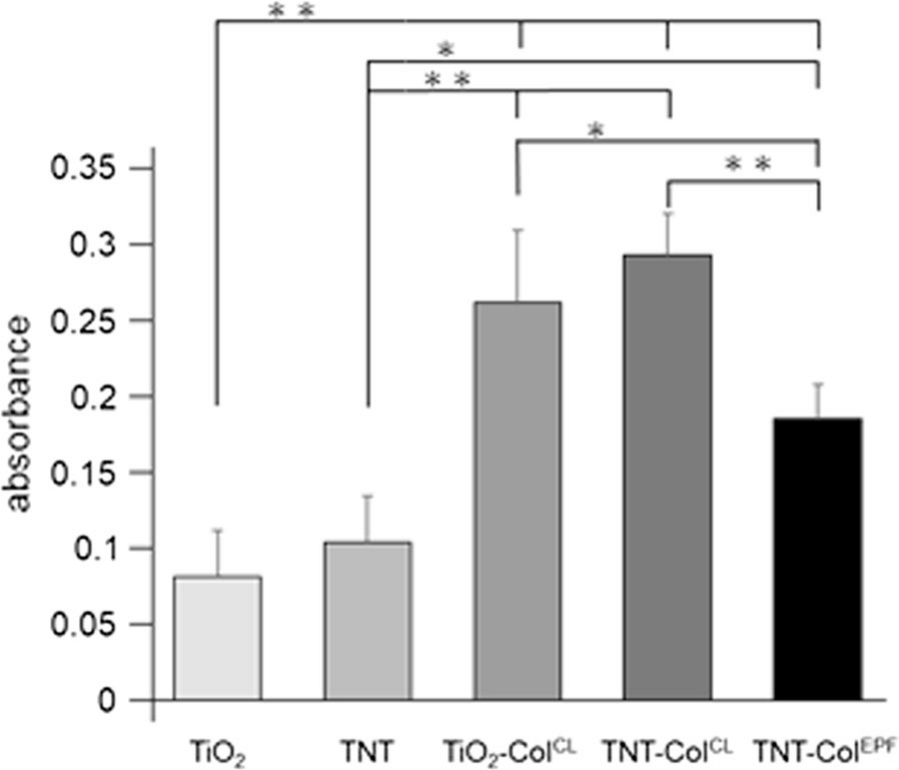
The comparison of relative cell amount on each group surface. Relative amount of epithelial cell on each group surface. Error bars means represent mean ± SD for n = 3, *P < 0.05, **P < 0.01

In addition, TNT–CoI^EPF^ showed limited numbers of epithelial cells in the comparison of relative cell amount on each group surface by CCK8 reagent (Fig. 9).

## Discussion

In this study, we aimed to fabricate sufficient of protrusion of CoI from TNT surface that would prime subsequent self-assembling of cell-secreted CoI. We hypothesized that the optimal depth of TNT should be around 100 nm which could support CoI perpendicularly and leave 200 nm of protrusion. Diameter and depth of TNT are controlled by voltage and duration. As the applied voltage increased, the diameter of TNT increased, resulting in a regularly aligned array of TNT with 67 nm-diameter on average. The total length is mainly determined by the duration of anodization. In this study, we found that 30 V and 3 h results in a uniform array of TNT measuring 50 to 100 nm in diameter with a depth of 2 μm on the titanium surface; however, this structure was brittle. Thus, a second step ultrasonication process in 30% H_2_O_2_ was carried out for 5 min to purposely remove the top layer of TNT, unveiling a glossy surface underneath with a uniform array of shallow TNT leaving a substantial depth of approximately 100 nm for CoI seeding (Fig. 1c). Indeed, several tens of nanometers of protrusion arrays of CoI were shown by AFM (Fig. 5). When compared to the AFM image of parallel-laying CoI (Fig. 5c1, c2, f1, f2) the orientation of TNT–CoI^EPF^ was perpendicular. The length of protrusion was shorter in the AFM images than the calculated length, likely because the probe of the AFM could not reach the bottom of the nanotube. The calculated length of the CoI protrusion should be sufficient as priming sites for collagen fibrillation.

Several technologies have been explored to alter the titanium surface with bioactive molecules [31, 32]. Biomolecules have been directly immobilized on titanium by passive adsorption or by covalent immobilization via a chemical linker. The binding site and orientation of macromolecules cannot be defined, due to multiple binding residues in the molecule. Electrophoretic deposition (EPD) is a traditional processing method in the ceramic industry that has been gaining interests for its potential in new material coating [33]. EPD deposits charged inorganic molecules with small molecular weight. The novelty and the key of success in the orientation-defined CoI fusion was the combination of EPD through polyacrylamide gel (PAGE). Triplet C-termini of CoI are negatively charged at the pH in the native PAGE system, therefore the triplet negative C-termini are towed towards anodic TNT through the gel mesh and reacts with hydroxyl group on the titanium surface. The triple carboxylic acid residues have two potential binding modes, monodentate and bidentate [34–36]. In addition to the C-term dentate bindings, acidic groups in the collagen chains also form dentate bond to the side wall of the TNT thus yielding the stable binding that we called EPF. Furthermore, CoI-dentate fusion will be enforced in collagen fibril/fiber that composes numerous dentate bindings on the TNT.

Perpendicular attachment of CoI in the TNT-holes and on the TNT-edges were shown by SEM (Fig. 4b) and AFM (Fig. 5e2). As perpendicular attachment of the gingiva-dental fibers block the downgrowth of junctional epithelium, perpendicular CoI on the TNT is anticipated to retain the vertical level of peri-implant epithelium at the abutment that connects crown and implant body. Moreover, when TNT–CoI^EPF^ is extended on the implant body, periodontal ligament like robust yet flexible suspension of implant could be obtained. Our future studies will focus on the biological reactions of TNT–CoI^EPF^ on connective tissue fibroblasts and alveolar bone osteoblasts.

In the FTIR-ATR spectrum (Fig. 6), the 1089 cm^−1^-peak corresponding to Ti–OH bonds between titanium and the hydroxyl groups of CoI was always high in TNT–CoI^EPF^ groups as compared to the other groups in either pre- and post-ultrasonic disruption. This indicated that high density (Pre-ultrasonication, Fig. 6a, b) and stable bond (Fig. 6c–f) of CoI was achieved by EPF. The binding stability of TNT–CoI^EPF^ was greater than that of the CL of PA, which is supposed to form mono-, bi- or tridentate bond to titanium [36]. Greater stability of the TNT–CoI^EPF^ driven Ti-CoI binding was most likely due to numerous dentate bonds with the acidic residues of CoI.

The frequency and maximum power of the ultrasonic disrupter which we used for the assessment of Col binding stabilities in this study was 20 kHz/250 W. In comparison, the frequency and maximum power of the ultrasonic generator typically used for dental scaling is 25 to 40 kHz/40 to 45 W. In this study, the assessment of Col binding stabilities was carried out at the maximum power of the ultrasonic disrupter, and the maximum power possessed of this equipment is much larger than the ultrasonic scaler for dental use. Therefore, it is conceivable that collagen attached on the titanium surface through EPF may possess sufficient resistant against the range of clinically relevant treatment procedures applied for prophylaxis and as anti-inflammatory therapy at the transmucosal dental implant.

In the epithelial cell attachment assay, on the titanium surfaces with CL-mediated parallel CoI coating on TiO_2_ and TNT, the cells adhered with spreading plasma membrane with pseudopod like stretching (Fig. 8c1, c2, d1, d2) suggesting the intercellular junction formation to complete the epithelial sheet. On the other hand, the cells sparsely localized and did not spread their plasma membrane (e1, 2). This most likely indicated that epithelial cells wrapped over the horizontal collagen layer as they do on subcutaneous connective tissue layer such as endothelial cells. The complex micro-sized surface substratum should influence epithelial cell behavior and slow epithelial cell migration substantially. TNT–CoI^EPF^ surface inhibited the initial adhesion of epithelial cells, and its perpendicular attachment of collagen fibers can prevent epithelial downgrowth if utilized on the dental implant surface.

Because of the osseointegration, titanium has been widely used for bone-anchored implants. On the other hand, current titanium has little affinity for robust soft tissue attachment. Robust immobilization of collagen either perpendicularly into TNT via EPF or horizontally on TiO_2_ via traditional CL. Horizontal collagen lamella may promote antibacterial epithelial sealing at the external interface of percutaneous implants where infection is common complication. On the other hand, we anticipate that the perpendicular collagen (TNT–CoI^EPF^) will be integrated with connective tissue gingival fibers that blocks epithelial downgrowth. Further anticipation could imagine the restoration of sensory nerve ending (SNE) at peri-implant ligament because: (1) periodontal and periosteum ligament is the place of SNE; and (2) ligamentous fibroblasts secrets inactive proforms of neutrophins and metalloproteases that activate the proneutrophins. Thus, soft-tissue integration on TNT–CoI^EPF^ implant may promote integration of sensory nerve endings into skin autografts which may be utilized in fingers, arms and legs prosthetics. We believe that our translational research will support future clinical development in various fields beyond dentistry.

## Conclusions

EPF achieved perpendicular fusion of CoI to TNT with several tens of nanometers of protrusion from the TNT ridge. Collagen self-assembly is anticipated from the TNT–CoI^EPF^ protrusion in the presence of fibroblasts or osteoblasts.

## Abbreviations

JE: junctional epithelium
BL: basal lamina
PDL: periodontal
TNT: titanium oxide nanotube
CoI: collagen type I
CoI/TNT: CoI into TNT
NH_4_F: ammonium fluoride
SEM: scanning electron microscopy
TiO_2_: titanium oxide
EPF: electrophoretic fusion
PA: phosphonic acid
CL: chemical linking
TGB: tris glycine buffer
AFM: atomic force electron microscopy
PFA: paraformaldehyde
TiO_2_–CoI^CL^: TiO_2_ linked collagen through CL
TNT–CoI^CL^: TNT linked collagen through CL
TiO_2_–CoI^EPF^: TiO_2_ fused collagen through EPF
TNT–CoI^EPF^: TNT fused collagen through EPF
FTIR-ATR: Fourier Transform Infrared Spectroscopy Attenuated Total Reflection
ANOVA: analysis of variance
EPD: electrophoretic deposition
PAGE: polyacrylamide gel
SNE: sensory nerve ending
